# Identification of Copy Number Aberrations in Breast Cancer Subtypes Using Persistence Topology

**DOI:** 10.3390/microarrays4030339

**Published:** 2015-08-12

**Authors:** Javier Arsuaga, Tyler Borrman, Raymond Cavalcante, Georgina Gonzalez, Catherine Park

**Affiliations:** 1 Department of Mathematics, University of California Davis, 1 Shields Avenue, Davis, CA 95616, USA; 2 Department of Molecular and Cellular Biology, University of California Davis, 1 Shields Avenue, Davis, CA 95616, USA; 3 Program in Bioinformatics and Integrative Biology, University of Massachusetts Medical School, Worcester, MA 01605, USA; E-Mail: tylerborrman@gmail.com; 4 Department of Computational Medicine and Bioinformatics, University of Michigan, Ann Arbor, MI 48109, USA; E-Mail: rcavalca@umich.edu; 5 Department of Mathematics, San Francisco State University, 1600 Holloway Avenue, San Francisco, CA 96132, USA; E-Mail: ginaglezi2000@gmail.com; 6 Helen Diller Comprehensive Cancer Center,University of California San Francisco, 1600 Divisadero Street, San Francisco, CA 94143, USA; E-Mail :Catherine.Park@ucsf.edu

**Keywords:** breast cancer subtypes, copy number aberrations, topological data analysis, TAaCGH

## Abstract

DNA copy number aberrations (CNAs) are of biological and medical interest because they help identify regulatory mechanisms underlying tumor initiation and evolution. Identification of tumor-driving CNAs (driver CNAs) however remains a challenging task, because they are frequently hidden by CNAs that are the product of random events that take place during tumor evolution. Experimental detection of CNAs is commonly accomplished through array comparative genomic hybridization (aCGH) assays followed by supervised and/or unsupervised statistical methods that combine the segmented profiles of all patients to identify driver CNAs. Here, we extend a previously-presented supervised algorithm for the identification of CNAs that is based on a topological representation of the data. Our method associates a two-dimensional (2D) point cloud with each aCGH profile and generates a sequence of simplicial complexes, mathematical objects that generalize the concept of a graph. This representation of the data permits segmenting the data at different resolutions and identifying CNAs by interrogating the topological properties of these simplicial complexes. We tested our approach on a published dataset with the goal of identifying specific breast cancer CNAs associated with specific molecular subtypes. Identification of CNAs associated with each subtype was performed by analyzing each subtype separately from the others and by taking the rest of the subtypes as the control. Our results found a new amplification in 11q at the location of the progesterone receptor in the Luminal A subtype. Aberrations in the Luminal B subtype were found only upon removal of the basal-like subtype from the control set. Under those conditions, all regions found in the original publication, except for 17q, were confirmed; all aberrations, except those in chromosome arms 8q and 12q were confirmed in the basal-like subtype. These two chromosome arms, however, were detected only upon removal of three patients with exceedingly large copy number values. More importantly, we detected 10 and 21 additional regions in the Luminal B and basal-like subtypes, respectively. Most of the additional regions were either validated on an independent dataset and/or using GISTIC. Furthermore, we found three new CNAs in the basal-like subtype: a combination of gains and losses in 1p, a gain in 2p and a loss in 14q. Based on these results, we suggest that topological approaches that incorporate multiresolution analyses and that interrogate topological properties of the data can help in the identification of copy number changes in cancer.

## 1. Introduction

Chromosome aberrations are large-scale structural changes of the genome that are commonly associated with cancer initiation and progression [1,2,3]. DNA copy number aberrations (CNAs), such as copy number gains and losses, are of particular interest, because they may respectively harbor oncogenes and tumor suppressor genes; hence, they have the potential to directly regulate cellular growth pathways (reviewed in [4,5,6]). CNAs that contain oncogenes or tumor suppressor genes are commonly known as driver aberrations; those that do not have functional implications are termed passenger aberrations. Genome-wide experimental detection of CNAs is achieved through microarray and/or DNA sequencing technologies [7,8,9,10,11,12]. Identification of driver CNAs, however, still remains a challenge [13,14,15,16,17,18,19]. One approach to identify such aberrations is through statistical supervised methods [19,20,21], which detect CNAs that are common and specific to a given cancer subtype or a cancer with specific clinical characteristics. Here, we propose a supervised method that identifies CNAs based on the topological properties of the aCGH profile. We call this method topological analysis of aCGH (TAaCGH).

TAaCGH associates a point cloud with each aCGH profile by means of a sliding window map [22,23] and uses the topological properties of the point cloud, obtained by standard techniques of persistence homology (reviewed in [24,25]), to identify regions of amplifications and deletions. Two properties differentiate our approach from other commonly-used supervised methods. First, TAaCGH performs a multiresolution segmentation of the data, similar to that of wavelets [26], and second, TAaCGH interrogates the topological properties of the data, rather than each of the independent clones or segmented regions of each patient profile.

In this study, we tested our approach by identifying CNAs that are specific to the molecular subtypes of breast cancer ([27,28], also reviewed in [29,30]), since it is known that different subtypes have different regulatory mechanisms and, in some cases, well-determined patterns of driver CNAs [31,32,33,34,35,36]. Several studies have also reported the association between CNAs and the evolution of the tumor or the response to treatment [7,37,38,39,40,41,42,43,44,45]. Therefore, an additional important aspect of CNA studies is the possibility to identify prognostic subgroups with different outcomes and/or responses to treatment within each gene expression subtype. We analyzed the data reported in [33] where CNAs associated with molecular subtypes Luminal A, Luminal B, ERBB2/HER2/NEU (denoted by HER2+) and basal-like were identified using the supervised algorithm called Supervised Identification of Regions of Aberration in aCGH (SIRAC) [21]. TAaCGH found all regions reported in the original publication for the Luminal A and HER2 subtypes and a new amplification at the location of the progesterone receptor gene (11q) in the Luminal A subtype. In the basal-like subtype, TAaCGH found all aberrations reported in [33], except 8q and 12q; these two CNAs were found upon removal of three patients that had exceedingly large copy number changes. Interestingly, TAaCGH also found 21 additional regions in the basal-like subtype, including a combination of copy number gains and losses in 1p, a gain in 2p and a loss in 14q. The Luminal B subtype only revealed specific CNAs when the basal-like subtype was removed from the control set. Under those conditions, TAaCGH found all CNAs reported in [33], except 17q and 10 new aberrations. Most of these newly-identified regions have been reported in other independent studies and were validated using an independent dataset [32] and/or using GISTIC [13]. We therefore suggest that the use of topological data analysis can help identify new aberrations in cancer.

## 2. Experimental Section

### 2.1. Simulation Data

Each simulated dataset consisted of 120 aCGH profiles, with 100 clones each. The 120 profiles were equally split between the test and control sets. The implementation of the simulated profiles followed the work of [21,46], where the copy number value of each clone was drawn from a Gaussian distribution of mean μ≠0 for clones inside an aberration and of mean μ=0 for clones outside any aberration. The standard deviation *σ* was constant for all clones in any given simulation. The mean value of an aberration was μ∈{-1,0.6,1}, the standard deviation of an aberration σ∈{0.2,0.5} and the length of an aberration (*i.e.*, number of clones) λ∈{2,3,5,10,20,50,75}. Simulations were repeated multiple times for each combination of parameters.

### 2.2. The Horlings Dataset

The dataset analyzed in this study was published by Horlings and colleagues [33]. Measurements of copy number changes were performed on microarrays containing 3.5 k Bacterial Artificial Chromosome (BAC) , P1-Derived Artificial Chromosome (PAC) DNA segments covering the entire genome with an average spacing of 1 Mb. Each BAC clone was spotted in triplicate on every slide (Code Link Activated Slides, Amersham Biosciences). Signal intensity measurements were captured using ImaGene Software (BioDiscovery, Inc.) and normalized by median print tip normalization. Intensity ratios (Cy5/Cy3) were log-transformed, and triplicate spot measurements were averaged. From a pool of 295 breast tumor specimens, 68 samples were selected to represent the most common molecular subtypes: Luminal A (n=21) and Luminal B (n=12), basal-like (n=21), HER2-enriched, also known as ERBB2/HER2/NEU, and denoted by (HER2+) (n=14). All samples contained 50% or more tumor cells. The raw data were not imputed; clone positions were outdated and, in some instances had, different clones associated with the same genomic position. Therefore, some preprocessing was required.

We found that the position of the clones reported in [33] did not match those in publicly-available databases. For instance, the position of the clone RP11 to 94L15n, which contains ERBB2, was reported to be at 35,065,321 bp on chromosome 17q in [33], but mapped to base pair position 37,812,853 in the ENSEMBL database. To address this issue, we remapped all clones according to the ENSEMBL database (built GRCch37). We found most clones located near or at the reported position; however, of the original 3277 clones in the Horlings study, we updated the position of 3021 clones and removed 256. We removed 122 clones that had no base pair information in the original publication or ENSEMBL. Ninety eight clones were in a chromosome different from that reported in the original publication, and eight clones were in the correct chromosome, but at a position located more than 5 × $10^{6}$ bps away from the position reported in ENSEMBL. Finally, we removed 28 clones that were in the correct chromosomes, but had inconsistent relative positions with respect to their immediate neighboring clones. We imputed missing values using the algorithm called locally weighted scatterplot smoothing (lowess) [47]. Entries of clones that were mapped to the same locations were averaged.

### 2.3. Detection of Focal Copy Number Aberrations Using TAaCGH

**Figure 1:**
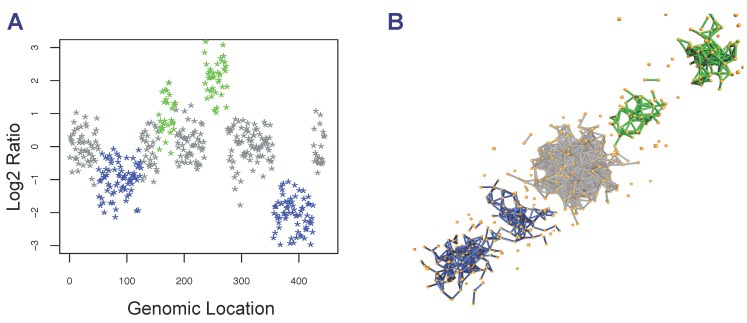
Generation of a point cloud from array CGH. (**A**) Idealized aCGH profile. Hypothetical copy number changes are colored in blue (losses) and green (gains). Non-significant changes are colored in silver. (**B**) Point cloud, with points connected by edges, associated with the aCGH profile using a sliding window approach

Here, we extend the method initially proposed in [22,48] to analyze microarray data (see the Conclusions Section for a detailed explanation of the new features reported in this work). For a chosen section of *m* copy number values, TAaCGH associates a point cloud in an euclidean n-dimensional coordinate system (*i.e.*, $R^{n}$), 1<n<m. We illustrate this association by building a point cloud in $R^{3}$ from a section of copy number values $\left\{y_{1},y_{2},...y_{m}\right\}$ (see Figure 1). Any three consecutive copy number values $\left\{y_{i},y_{i+1},y_{i+2}\right\}$ naturally define a point in $R^{3}$ with coordinates $\left(y_{i},y_{i+1},y_{i+2}\right)$ (*i.e.*, the first log ratio value, $y_{i}$, is assigned to the x coordinate of the point in the point cloud; the second log ratio value, $y_{i+1}$, is assigned to the y coordinate of the point; and the third log ratio value, $y_{i+2}$, to the z coordinate of the point). This algorithm is well defined everywhere, except when i=m-1,m (*i.e.*, the last two copy number values of the section), since the third coordinate of the point in the cloud (in $R^{3}$) is not defined when i=m-1, and neither the second nor the third coordinates are defined when i=m. To solve this problem, TAaCGH completes the missing entries by considering the first values of the section (*i.e.*, $\left\{y_{1},y_{2}\right\}$). In order to represent the entire section of copy number values as a single point cloud, TAaCGH uses a sliding window approach. Therefore, the point cloud generated by a section of consecutive copy number measurements $\left\{y_{1},y_{2},...y_{m}\right\}$ is $\left\{\left(y_{1},y_{2},y_{3}\right),\left(y_{2},y_{3},y_{4}\right),\left(y_{3},y_{4},y_{5}\right),...\left(y_{n-1},y_{n},y_{1}\right),\left(y_{n},y_{1},y_{2}\right)\right\}$. In Figure 1A, an idealized profile with gains (green), no changes (silver) and losses (blue) is shown. The associated point cloud in $R^{3}$ is shown in Figure 1B with the points connected by edges (see below for an explanation of the meaning of the edges). A number of features can be noticed when representing the data as a point cloud. First, the associated point cloud has an elliptical shape, because consecutive copy number values are correlated. In fact, when TAaCGH was applied to gene expression profiles, we observed that the associated clouds were spherical due to the lack of correlation between expression values of consecutive genes along the genome [48]. Second, consecutive gains are mapped to the octant with all positive values, consecutive losses to the octant with all negative values, and values containing combinations of positive and negative values are mapped to the other octants. Third, the higher the absolute value of the gain or loss, the further the corresponding points in the point cloud will be from the origin. Consequently, the noise in the data is mapped near the origin of the coordinates.

The next step in the algorithm is to build a filtration of Vietoris-Rips simplicial complexes. The goal of this step is to build a segmented picture of the data from which topological properties can be inferred. Intuitively, a Vietoris–Rips simplicial complex is a generalization of a graph that is built as follows: for a point cloud in $R^{n}$ and a fixed small number *ϵ* (called the filtration coefficient), one defines an edge between two points in the cloud if the euclidean distance between the two points is less than or equal to *ϵ*. If n≥2, then solid triangles are also part of the Vietoris–Rips simplicial complex, and a solid triangle between three points is included in the complex if the three points are connected by edges. This process is also valid in higher dimensions and is generalized by adding tetrahedra and higher dimensional minimal convex sets. It is evident that for any two values $\epsilon_{1}<\epsilon_{2}$, the associated simplicial complexes $S_{1},S_{2}$ satisfy $S_{1}\subset S_{2}$. Therefore, if one lets the filtration coefficient systematically increase $\epsilon_{1}<\epsilon_{2}<\epsilon_{3}<...<\epsilon_{p}$, one obtains a filtration of simplicial complexes $S_{1}\subset S_{2}\subset S_{3}\subset ...\subset S_{p}$ (see [24,25] for a detailed description). We propose that in the analysis of aCGH data, the associated filtration can be viewed as a continuous segmentation process that assigns the same copy number value to clones whose copy number value difference is less than *ϵ*. In other words, when two points in the point cloud connect, they become part of the same element in the simplicial complex. This identification of points in the point cloud can be interpreted as a segmentation step, where the clones generating the points in the point cloud are assigned the same copy number value.

The key property of this representation of the data is that it allows us to perform association studies between the phenotype of interest and the topological properties of the simplicial complexes. In this work, we have done so for the number of connected components (called the zeroth Betti number and denoted by $\beta_{0}$), a topological property that measures the number of detached subsets that make up a dataset. We chose to start with the number of connected components, because, as proposed in [22] and illustrated in Figure 1, the value of $\beta_{0}$ helps identify CNAs. Calculations of $\beta_{0}$ were done using the software jPlex [49].

By considering the values of $\beta_{0}$ across the filtration, one obtains a function $\beta_{0}\left(\epsilon \right)$ that relates the number of connected components to each distance *ϵ*. Furthermore, given two sets of patients (test and control), it is natural to compute the average value of $\beta_{0}$ for each value of *ϵ* for the control and test set separately and to associate a *p*-value with the difference between the two measurements. TAaCGH identifies significant differences in $\beta_{0}$ with differences in copy number values between the two populations.

**Figure 2:**
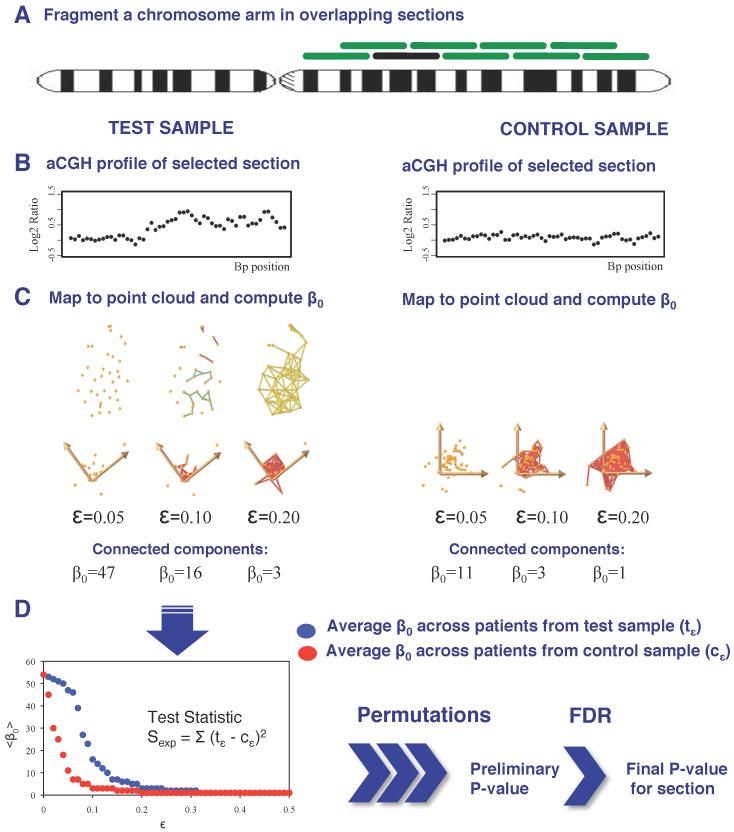
Topological analysis of array CGH. (**A**) The ideogram of chromosome 1 and the overlapping sections to be analyzed (in green). One section (black) is selected and analyzed as illustrated in the following panels. (**B**) Array CGH profiles of the chromosome section for two patients (test and control) taken from [7]. (**C**) aCGH data are mapped using a sliding window algorithm to the Euclidean two-dimensional coordinate system, $R^{2}$, and a filtration of simplicial complexes is generated using values of the filtration parameter ϵ=0.05,0.10,0.20. (**D**) For each *ϵ*, the average $\beta_{0}$ is computed for both the test sample ($t_{\epsilon }$, blue) and the control sample ($c_{\epsilon }$, red) and plotted into one single graph from which the test statistic $S_{exp}$ is calculated. The *p*-value is calculated using a permutation test and corrected using the false discovery rate (FDR), because of the multiple sections being tested.

Figure Figure 2 presents the algorithm for detecting sections of CNAs using TAaCGH. (A) shows the ideogram of a chromosome in which the section to be analyzed has been highlighted in green. (B) shows the aCGH profiles for one of the sections for two patients taken from [7]. The profile on the left-hand side has an amplification, while the one on the right has no copy number changes. The sliding window approach described above will create a point cloud of copy number values in $R^{n}$ for each profile. Figure 2C shows the corresponding point clouds and the one-dimensional Vietoris-Rips simplicial complexes for the two profiles (for n=2). The example on the left, corresponding to the profile with the copy number gain, clearly shows two large connected components. One component is located at the origin (red) and accounts for all clones with small copy number values. The second component, away from the origin (in yellow), contains the copy number values corresponding to the amplification. The point cloud on the right shows only one connected component at the origin. Hence, in TAaCGH, each patient is not only represented by his or her associated point cloud, but by his or her corresponding filtration, from which the topological invariants can be calculated for each value of *ϵ*.

For a group of patients of size *m*, TAaCGH calculates the value of $\beta_{0}$ for each patient *i* and the value of *ϵ* (denoted by $\beta_{0}\left(i,\epsilon \right)$) and then computes $<\beta_{0}\left(\epsilon \right)>=\frac{1}{m}\sum \beta_{0}\left(i,\epsilon \right)$ for i=1,…,m, the average value of $\beta_{0}$ across all patients in the population. Since one obtains one $<\beta_{0}\left(\epsilon \right)>$ for each *ϵ*, this average value naturally defines a function on *ϵ*, which we denote by $<\beta_{0}>$. Based on the construction described, two populations of patients can be compared by identifying significant differences between their associated $<\beta_{0}>$ curves, which represent copy number changes present at one population and not the other. Figure 2D shows examples of $<\beta_{0}>$ curves for two samples: a test sample (in blue) containing profiles similar to the sample profile with the aberration and a control sample (in red) with no aberration at that particular location. The shape of the $<\beta_{0}>$ curves can be easily interpreted. For very small distances, every data point will contribute one component. As the distance *ϵ* increases, points that are at a distance less than *ϵ* connect, decreasing the number of components. Eventually, for a sufficiently large value of *ϵ*, all points connect to form a single connected component.

To test statistically-significant differences between the test and control $<\beta_{0}>$ curves, we used the sum of the squares of the differences in average $<\beta_{0}>$ across all values of *ϵ*, *i.e.*, $S_{exp}=\sum {\left(t_{\epsilon }-c_{\epsilon }\right)}^{2}$ for ϵ=0,…,K, where $t_{\epsilon }$ and $c_{\epsilon }$ are the average number of connected components for the test set and the control set, respectively, and where *K* is the smallest number, such that $t_{\epsilon }=c_{\epsilon }=1$. The null hypothesis tested was $S_{exp}=0$. In other words, there was no difference between the test and control $<\beta_{0}>$ curves. We defined the referent distribution using a standard permutation test between genotypes (*i.e.*, aCGH profiles) and phenotypes (*i.e.*, tumor subtype). Since this selection of *p*-values assigns one *p*-value per section, we corrected the final *p*-value using the false discovery rate (FDR) [50,51].

When analyzing tumor data, the test population consisted of a specific subtype, and the control population consisted of the remaining subtypes. Hence, both populations, test and control, had aberrations. In some cases, we found that the control $<\beta_{0}>$ curve had larger values than the test $<\beta_{0}>$ curve, suggesting that the control set had more CNAs at that particular location. These cases were not considered in our analysis, since they provided information of the control and not the test dataset.

### 2.4. Determining Significance of Specific Clones

TAaCGH determines a chromosome section that contains significant changes, but it does not identify specific clones with significant copy number changes or whether the change is an amplification or a deletion. To narrow down the search for clones with significant copy number changes and to identify whether copy number changes were gains or losses, we compared the mean copy number value at each clone between the control and the test population. Significance was assessed using a permutation test. Since both the test and control populations have CNAs, sometimes at identical locations, some chromosome arms were identified as significant using TAaCGH, but no clones were found significant. These few regions were still considered as significant, but classified as undetermined.

### 2.5. Detection of Full-Length Arm/Chromosome Section Aberrations

The topological approach of TAaCGH is designed to measure relative changes in copy number between a clone and its neighbors and, therefore, does not account for large-scale chromosome aberrations, such as full arm amplifications or deletions. To detect large-scale chromosome aberrations, we extended the topological method by measuring significant displacements of the center of masses of the point clouds. More specifically, the center of mass of the point cloud associated with each chromosome arm was calculated for each patient and averaged over all patients belonging to any given category. A *p*-value was assigned to the difference between the average value for the center of masses for both populations using the same permutation approach described above. We not only tested for significant differences between test and control, but also for significant displacements of the center of masses of the test population from the origin. This second test allowed us to drop those cases where the significant displacement was driven by the control population.

### 2.6. Validation of the Experimental Results

We took three different approaches to validate our findings: (1) we compared our findings to those reported in the original publication [33], using SIRAC [21], and with other related publications [31,36,52]; (2) those that were found in our study, but not in the original paper [33], were tested in a second dataset [32]; and (3) we performed an independent analysis of the original data using the program GISTIC [13]. To apply GISTIC to the dataset analyzed in this study, we first segmented each profile using circular binary segmentation [53]. After segmentation, GISTIC found the aberrations per patient, and we computed the percentage of patients of a given subtype with each aberration. Since the sample size for some subtypes was small, we reported only those aberrations that were present in at least 35% of the patients (the full table of aberrations detected by GISTIC can be found in the Supplementary Material). While we expected to have an overall agreement with GISTIC, we also expected GISTIC to detect extra aberrations, because TAaCGH is designed to detect CNAs that are specific to a given subtype.

## 3. Results and Discussion

In the following section, we present our results using TAaCGH. Simulation results, obtained using the methods described in Section 2, are all presented in Section 3.1, and analysis of aCGH data are presented in Section 3.2.

### 3.1. Simulation Results

We performed simulation studies to optimize the value of the parameters in TAaCGH. First, we determined the size of the window (dimension of the point cloud) for analyzing the data. Second, we estimated the sensitivity and specificity of TAaCGH for a fixed window size, and third, we estimated the length of each chromosome section to be analyzed (green bars in Figure 2A). Lastly, we tested the performance of the TAaCGH of regions containing aberrations in the test and the control sets.

#### 3.1.1. Window Size

First, we investigated the role of the window size (or, in other words, the dimension in which the point cloud is embedded). The number of clones in the simulated section was 50 (instead of 100); the length of the aberrations (*i.e.*, the number of clones in the aberration) λ=2,3,5,10,20; the mean value of the aberration μ=-1,1 and 0.6; and the standard deviation σ=0.2 and 0.5. We considered all possible combinations of (λ,μ,σ) for window sizes D=2,5,10,15,20,35,50 and obtained a *p*-value for each experiment. Each experiment was repeated 84 times. The vectors of *p*-values obtained by combining all of this information were used to compute correlations across dimensions. Table 1 shows the results. We observed that all values were highly correlated (≥0.84). Furthermore, all dimensions were consistent in their significance assignments (results not shown). We concluded that using D=2 was enough to detect aberrations and performed the remainder of the studies at this dimension.

**Table 1:** Correlation among *p*-values across dimensions. Each entry in the table shows the correlation between the *p*-values obtained for the dimensions indicated in each row and column.

Dimensions	2	5	10	15	20	35	50
2	1	0.89	0.92	0.95	0.93	0.93	0.89
5		1	0.91	0.92	0.92	0.91	0.85
10			1	0.95	0.94	0.95	0.84
15				1	0.98	0.97	0.93
20					1	0.99	0.91
35						1	0.93

#### 3.1.2. Sensitivity and Specificity of TaACGH

**Figure 3:**
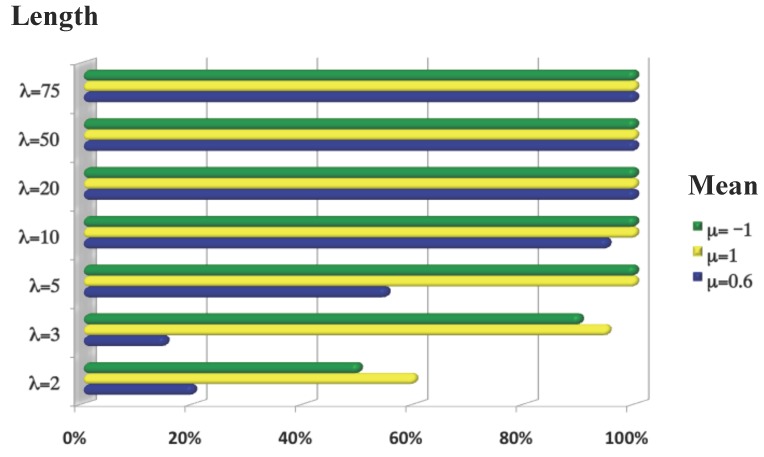
Sensitivity of TAaCGH using simulations. The chart shows the sensitivity of TAaCGH to the length and the mean value of the aberration λ and *μ*, respectively. Each row represents the different values of λ, and each color represents a different value of *μ*.

To test the sensitivity and specificity of our method, we performed simulations analyzing the parameters that define the aberrations. Sensitivity was estimated with aberration parameters μ=-1,0.6,1, σ=0.5 and λ=2,3,5,10,20,50,75. Specificity was estimated by simulating cases and controls with μ=0 and σ=0.5 and obtained a 100% success rate. Each experiment was repeated at least 20 times for each combination of parameters; *p*-values were corrected by FDR. Results for sensitivity are shown in Figure 3. This figure shows that TAaCGH has excellent sensitivity when a segment has three or more consecutive copy number changes for both μ=1 and μ=-1. This value sharply decreases when μ=0.6 and λ<5.

#### 3.1.3. Size of the Chromosome Section

Next, we analyzed the effects of the size of the section under analysis. One expects that very large sections will produce poor results, since different aberrations may form topologically-indistinguishable point clouds. For example, two sections with one amplification each with the same mean, but at different locations produce identical point clouds. On the other hand, clouds with a small number of points are not expected to be very informative. We therefore computed the sensitivity and specificity when the point clouds were made of 20,50,80 and 100 points and the following parameters D=2, μ=1, σ=0.5 and λ=2. Each experiment was repeated 100 times. We observed that the larger the point cloud, the worse the sensitivity decreasing from 100% for 20 points to 62% for 100 points. Specificity was 100% for all cases.

#### 3.1.4. Performance of TAaCGH when Both Control and Test Population Have Overlapping Aberrations

**Figure 4:**
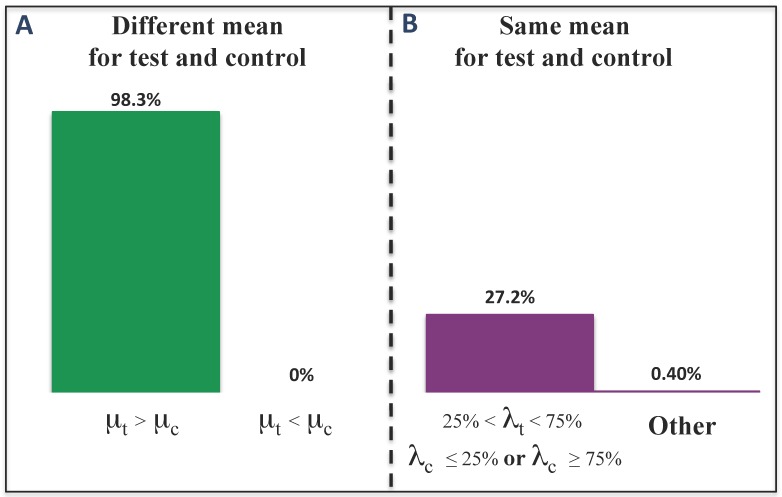
Sensitivity and specificity of TAaCGH on sections with copy number aberrations (CNAs) in both the test and control groups. The chart shows the sensitivity of TAaCGH to the length and the mean value of the aberrations λ and *μ*, respectively. (**A**) Sensitivity when the mean for the test ($\mu_{t}$) and the mean for the control ($\mu_{c}$) are different. If the mean for the test group is larger than the one from the control, sensitivity was 98.3%. If, on the other hand, the mean from the control was larger than the test group, TAaCGH did not detect the aberration in the test group; (**B**) Poor sensitivity when both the test group and the control group have an aberration in the section with the same mean. When the length for the aberration in the test group ($\lambda_{t}$) has a medium size, that is $25\%<\lambda_{t}<75\%$, from the size of the section, the sensitivity is 27.2%; otherwise, it will be close to 0%.

In the last simulation study, we analyzed the performance of TAaCGH when both the control and the test set had a CNA at the same location. We considered a total of 60 patients in each category with fixed section size (= 20), standard deviation σ=0.5 and dimension D=2. The values of *μ* and λ ranged from {0.6,1,-1} to {5,10,15}, respectively. For clarity, we denoted $\mu_{c}$ and $\mu_{t}$ the mean values of the CNA in the control and test group and by $\lambda_{c}$ and $\lambda_{t}$ the values of the length of the CNA for both sets. Results of our simulations are shown in Figure 4.

As illustrated in Figure 4A, TAaCGH identified the aberration in the test set in almost all cases when $\mu_{t}>\mu_{c}$, but never when $\mu_{c}>\mu_{t}$, since those cases produced $<\beta_{0}>$ curves in which the control set had higher values than the test set. When $\mu_{t}=\mu_{c}$, the performance was poor, as indicated in the bar chart on Figure 4B, almost independently of the length of the aberration.

### 3.2. Results for Breast Cancer Subtypes

**Figure 5:**
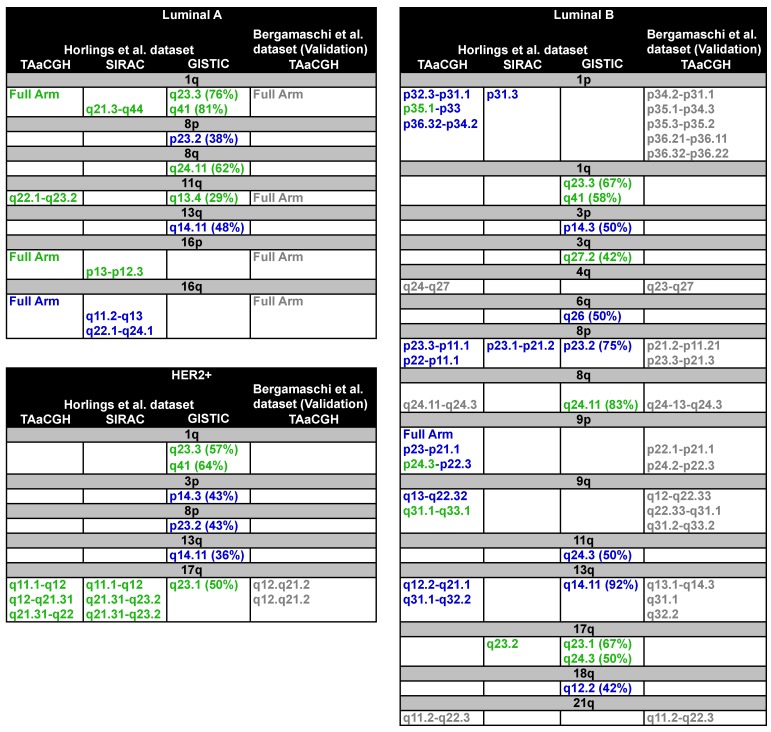
Summary results for Luminal A, Luminal B and HER2+ subtypes. The three panels show significant aberrations found by TAaCGH, SIRAC or GISTIC. Only results for TAaCGH that were validated in an independent dataset are shown. The frequency cut-off in GISTIC was 35%. Amplifications are denoted in green, deletions in blue and undetermined in grey. Sections with both colors (blue and green) contained combinations of amplifications and deletions. Arms validated using TAaCGH on a second dataset [32] are also color coded in grey.

**Figure 6:**
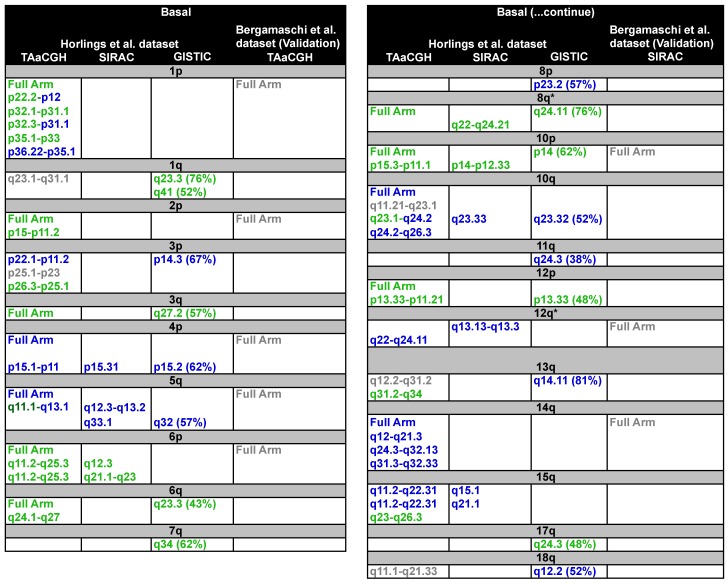
Summary results for basal-like subtype. The two panels show significant aberrations found by TAaCGH, SIRAC or GISTIC. Only results for TAaCGH that were validated in an independent dataset are shown. The frequency cut-off for GISTIC was 35%. Amplifications are denoted in green, deletions in blue and undetermined in grey. Sections with both colors (blue and green) contained combinations of amplifications and deletions. Arms validated using TAaCGH on a second dataset [32] are also color coded in grey. * Significance when outliers were removed from the control set.

Samples were divided into subtypes; each subtype was considered separately as a test set, using the remaining subtypes as the control. In our analysis, chromosome arms were subdivided into overlapping sections; each section contained 20 clones, and any two consecutive clones overlapped 10 clones (Figure 2A). $<\beta_{0}>$ curves were calculated for the test and the control populations for a window size of n=2. The obtained *p*-values were then corrected for multiple testing using FDR (Figure 2D). Results obtained for the entire study are shown in Figure 5 and Figure 6 and in Supplementary Files S1–S8. Each panel in Figure 5 and Figure 6 shows the specific subtype with the location of the significant aberrations found by TAaCCH, SIRAC and GISTIC. Green entries mean amplifications, blue deletions and grey undetermined. Aberrations validated on an independent dataset are also color-coded in grey. Supplementary Files S1–S6 show the statistics for each of the subtypes; S7 shows the results of the analysis using GISTIC; and S8 shows patients that were removed from the control set due to their large copy number values (*i.e.*, outliers).

#### 3.2.1. Analysis of Luminal Subtypes

**Figure 7:**
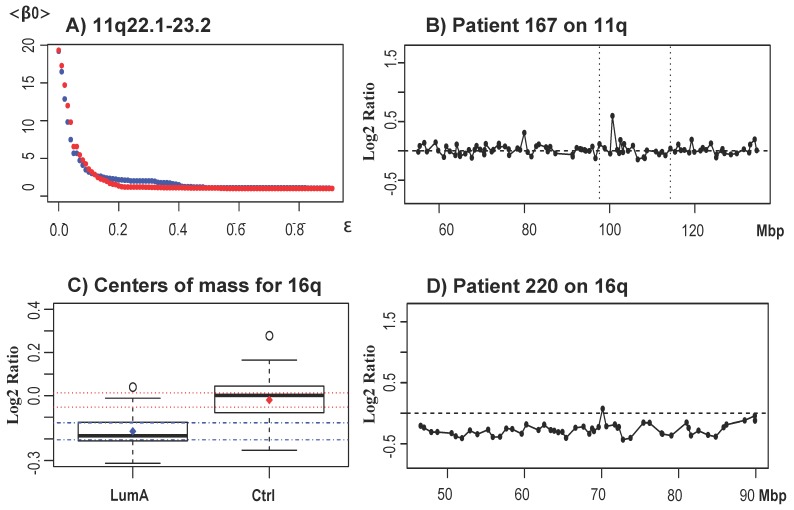
Results for the Luminal A subtype. (**A**) $<\beta_{0}>$ curves for the Luminal A significant region in 11q. The blue curve corresponds to the Luminal A subtype and the red curve to the control set; (**B**) A characteristic profile of a Luminal A patient with an amplification at 11q22.1 (100,641,187 bp) in the significant region 11q22.1 to q23.2 (vertical black bars); (**C**) A box plot of the center of masses of the Luminal A subtype *vs*. the control. The diamond in the center of the box shows the average, and the horizontal dotted lines represent the confidence intervals. Blue confidence intervals correspond to the Luminal A subtype and red to the control set; (**D**) The profile of a patient with a deletion of the entire chromosome arm 16q.

We analyzed Luminal A and B subtypes separately. The Luminal A subtype clinically is associated with the most favorable disease prognosis among the molecular subtypes. It is commonly estimated by pathologic characteristics, namely the estrogen receptor (ER) +, progesterone receptor (PR) + and ERBB2/HER2 (−), with low proliferation and with a low number of chromosome aberrations. Commonly-observed aberrations in the Luminal A subtype include 1q, 8q, 8p, 11q gain, 16p and 16q loss [31,33,36,52,54]. Our topological analysis found a single significant region 11q22.1 to q23.2. Within this region, only the clone at position 100,641,187 was significantly amplified. Figure 7A shows the corresponding $<\beta_{0}>$ curves and Figure 7B shows an example of a Luminal A aberrant profile at 11q.

Analysis of the displacement of the centers of mass for whole chromosome arms found 1q, 16p and 16q to be significant. Figure 7C shows the box plots for the displacements of the center of masses for chromosome arm 16q (for the Luminal A subtype and for the control set) together with a representative profile of a whole chromosome arm deletion for 16q (Figure 7D). The three arms 1q, 16p and 16q were significant in the original study by Horlings, as well as in many other studies [32,35,36,52,55,56] and were also validated when applying TAaCGH to the data published in [32]. Interestingly GISTIC identified CNAs in 1q and 11q, as well as CNAs in 8p, 8q and 13q, but failed to identify 16p and 16q. Based on our simulations, we expected TAaCGH not to be able to identify all aberrations detected by GISTIC, since they were common to different subtypes. In this particular case, 8p was common to 51% of all patients across subtypes; 8q was common to 65%; and 13q was common to 63% (see Supplementary File S7).

Next, we analyzed the Luminal B subtype. Luminal B patients are characterized by ER+, HER2− or HER2+, but have higher proliferation rates than Luminal A. Luminal B subtype cancers have generally worse prognosis than Luminal A. CNAs commonly observed in Luminal B patients include gains of 1q, 8p12 to p11, 8q, 11q13 to q14, 17q and 20q and losses in 1p, 8p, 13q, 16q, 17p and 22q (reviewed in [29,54]; see also [31,32,36,57,58]). Our topological analysis did not find any significant aberrations associated with this subtype. The displacement of the center of masses found 12q, but this arm was not confirmed on the validation dataset. A deeper analysis of the Luminal B set revealed that the initial significance was driven by Patient 378 with extremely large copy number values (see Supplementary File S6) and by a small cohort of Luminal B patients (n=12). Supporting this conclusion is the fact that we did not find any significant aberrations after removing Patient 378.

**Figure 8:**
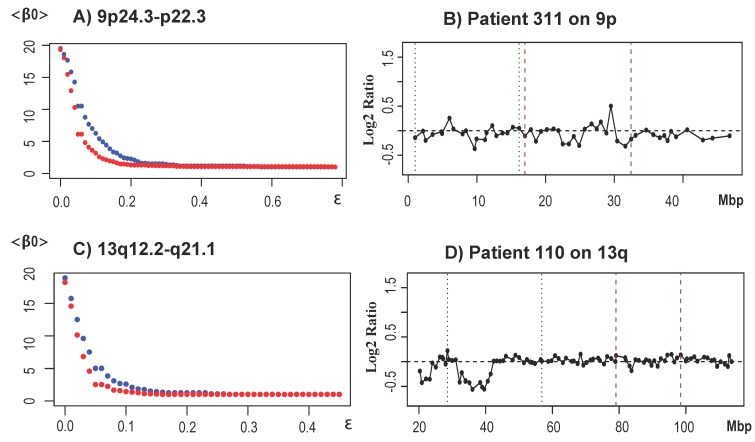
Selected results for the Luminal B subtype. (**A**) $<\beta_{0}>$ curves for Luminal B in significant region 9p24.3 to p22.3. The blue curve corresponds to the Luminal B subtype and the red curve to the control set (not including the basal-like patients); (**B**) A characteristic profile of a Luminal B patient from 9p with deletions at 9p21.3, 9p22.1 and 9p23.9 (23.4, 18.6, 9.7 Mbp), as well as an amplification at 9p24.1 (6 Mbp) in the significant regions 9p23 to p21.3 (vertical brown dashed lines) and 9p24.39 to 22.3 (vertical black dotted lines); (**C**) $<\beta_{0}>$ curves for Luminal B in significant region 13q12.2 to q21.1. The blue curve corresponds to the Luminal B subtype and the red curve to the control set from which the basal-like patients were removed; (**D**) A characteristic profile of a Luminal B patient from 13q with deletions at 13q12.2 to q14.11 (32.2 to 41.5 Mbp) in significant region 13q12.2 to q21.1 (vertical black dotted lines).

We performed several tests to understand why TAaCGH did not find the regions 1p31.3, 8p21.2 to p23.1 or 17q23.2 reported in the original study [33] and in other studies [31,32,36]. Since Luminal B is known to be a rather heterogeneous subtype and some of its CNAs may be shared across different subtypes [54], we hypothesized that some of these regions could be aberrant in more than one subtype. We therefore tested if the removal of specific subtypes from the control set would change our *p*-values. Upon removing the basal-like subtype from the control set, we found the significance of the regions 1p36.32 to p31.1, 4q24 to q27, 8p23.3 to p22, 8p22 to p11.1, 8q24.11 to q24.3, 9p24.3 to p21.1, 9q13 to q22.32, 9q31.1 to q33.1, 13q12.2 to q21.1, 13q31.1, 13q32.2, 21q11.2 to q22.3. TAaCGH identified specific amplified/deleted clones for the indicated significant regions, except for chromosome arms 4q, 8q and 21q, suggesting that these arms contain heterogeneous regions of CNAs that are common to several subtypes. Since 8p23.1 to p21.2 and 1p31.3 were reported in [33], they were not analyzed any further. Figure 8 shows the $<\beta_{0}>$ curves and patient profiles for regions 9p24.3 to p22.3 ((A) and (B)) and 13q12.2 to q21.1 ((C) and (D)). Figure 8B,D shows representative profiles that contain the significant CNAs reported in Table 2. To test whether these newly-identified regions were specific to [33], we performed a validation test using the dataset published in [32] and also compared it to those results obtained by GISTIC. All of the regions were validated in [32], although in some cases, small fragments within significant regions were not validated. This effect was most likely due to the higher resolution of the array. For instance within 1p36.32 to 31.1 we validated regions 1p36.32 to p36.22, 1p36.21 to p36.11, 1p35.3 to p35.2, 1p35.1 to p34.3, 1p34.2 to p31.1. Most of the regions, or regions in close proximity, not reported by [33], but found in our study, have been reported in other studies. For instance, 8p22 to p11, 8q, 9p, 9q, 13q and 21q have been reported as either focal or whole arm aberrations in [31,32,36,38,45,59,60,61,62,63]. Significant clones for 8p are shown in Supplementary File S6. In agreement with TAaCGH, GISTIC also detected 8p, 8q and 13q (all common to more than 75% of the patients in the Luminal B subtype), but failed to detect 1p, 4q, 9p, 9q, 21q. On the other hand, GISTIC detected 1q, 3p, 3q, 6q, 11q, 17q and 18q. As expected, TAaCGH did not detect these CNAs, because they were common to the test and control set. For instance, 1q23.3 was common to 68%, and 1q41 was common to 60% of the patients across all subtypes. The remaining chromosome arms were shared by different amounts of patients, ranging from 25% for 6q to 43% for 3p. It may seem that 25% is a small number of patients; however, when we looked at the distribution of patients across subtypes, we found that half of these patients were in the Luminal B set (test set) and the other half in the HER2+ (control set; see the distribution of aberrations in the Supplementary File S7).

The positions of the significant clones within the identified significant regions were identified next and are presented in Table 2.

Lastly, we removed the HER2+ subtype from the control dataset, but no significant aberrations were detected. In conclusion, upon removal of the basal-like dataset from the control group, our study found all regions reported in [33], except 17q23.2, and found 10 other regions that were also validated in [32], three of which were validated by GISTIC.

**Table 2:** Chromosome aberrations detected by TAaCGH in the Luminal B subtype.

Chromosome Arm	Cytoband	Location of Aberration	Gain/Loss	Location of Neighboring Clones
1p	36.32	3,225,674	loss	3,225,674 to 4,577,827
	36.22	10,154,043	loss	9,161,350 to 11,561,620
	36.22	11,561,620	loss	11,064,731 to 11,844,141
	36.22	12,429,632	loss	11,844,141 to 14,639,539
	36.21	15,553,913	loss	14,639,539 to 17,268,504
	36.12	23,338,991	loss	22,032,838 to 24,480,408
	36.11	27,703,779	loss	27,359,936 to 27,856,736
	34.3	35,105,342	gain	34,380,537 to 36,832,678
	34.3	38,492,287	loss	38,278,515 to 39,536,339
	33	50,571,477	loss	49,145,731 to 50,823,002
	31.33 to 31.1	65,599,782 to 70,103,164	loss	64,435,137 to 70,406,779
	31.1	76,869,240	loss	75,463,114 to 78,005,143
9p	24.1	6,004,718	gain	4,922,574 to 6,576,990
	23	9,668,611	loss	8,409,615 to 9,943,073
	22.1	18,590,957	loss	17,850,221 to 19,321,518
	21.3	23,387,562	loss	22,490,595 to 24,101,721
9q	21.11	71,549,399	loss	71,129,855 to 72,258,752
	21.31	83,762,927	loss	82,953,907 to 84,780,238
	22.2	93,147,096	loss	92,847,611 to 94,131,899
	31.1	106,545,018	gain	98,981,704 to 107,489,797
13q	12.2 to 31.1	32,170,305 to 41,470,434	loss	31,017,797 to 51,431,527
	31.1	79,057,929	loss	56,818,886 to 81,814,181
	31.1	83,138,436 to 83,695,803	loss	81,974,786 to 84,869,575
	31.1	84,869,575 to 87,444,574	loss	83,695,803 to 88,048,738

#### 3.2.2. Analysis of the ERBB2/HER2/NEU (HER2+)-Enriched Subtype

**Figure 9:**
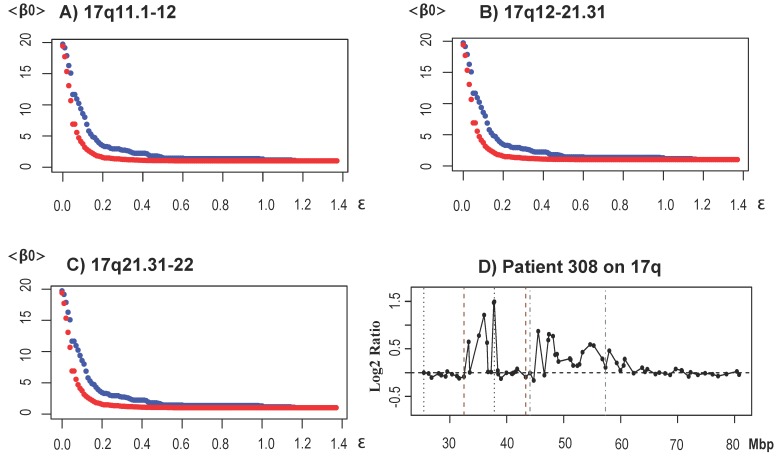
Results for the HER2+ subtype. (**A**) to (**C**) show $<\beta_{0}>$ curves for the regions indicated. The graphs show $<\beta_{0}>$ for the HER2+ patients in blue and the control set in red. All three regions indicated showed significance. (**D**) A profile with the significant regions in between dotted vertical lines. ERBB2 is located near the position $4\times 10^{7}$.

Next, we analyzed the ERBB2/HER2/NEU-enriched subtype. In HER2+ patients, overexpression of HER2 is commonly regulated by an amplification of the chromosome region containing the gene ERBB2 [64,65]. Only regions 17q11.1 to q12, 17q12 to q21.31 and 17q.21.31 to q22 were significant, and the corresponding $<\beta_{0}>$ curves are shown in Figure 9A–Figure 9C.

Our study, in agreement the the original study [33] and others [32,36,66], found the location of ERBB2 (cytobands 17q11.1 to q12). Furthermore, in agreement with the study by Horlings, we found a region extending beyond ERBB2 and containing cytobands 17q21.2 to q22. Significant clones were located between base pair positions 37,258,265 to 38,428,492 and 48,120,796 to 48,817,562. Figure 9D shows the profile of a patient with amplifications at the significant regions.

These results were also validated in [32]. In particular, our validation study confirmed a significant amplification encompassing regions 17q12 to 17q21.2. GISTIC was consistent with these findings and also found that more than 35% of the patients in the HER2+ subtype had aberrations in 1q, 3p, 8p and 13q. Similarly to our previous remarks, these CNAs were not detected by TAaCGH, because they were common to a large percentage of the patients in the study (>43%).

#### 3.2.3. Results for the Basal-Like Subtype

The basal-like subtype is the most heterogeneous subtype and includes those that are termed triple negative, indicative of the absence of ER, PR and HER2 expression. This subtype is generally associated with the worst prognosis of the subtypes, perhaps in part due to lack of targeted therapies. Consistent with this heterogeneity, we found the basal-like subtype to have the highest number of CNAs in a total of 29 different regions. We present the significant sections of the genome found in our study according to our validation results

i. CNAs in agreement with those reported in the original study

We found the statistical significance of all arms reported in [33] (*i.e.*, 4p, 5q, 6p, 10p, 10q and 15q) with the exception of 8q and 12q. From these, the only arms that were not significant by the center of masses were 12q and 15q. Our significant regions did not always match the regions reported in [33]. These discrepancies between both studies were either because of the updated position of the clones in our study or because TAaCGH found regions containing those reported in [33]. For example, we found the significance of the region 4p15.1 to p11 instead of the reported region 4p15.31. Region 4p15.31, however, became significant when the clones were placed following Horlings’ original position. Another example was the loss of 5q12.3 to q13.2, where we found an overlapping region expanding from 5q11.1 to 5q13.1. We did not detect 8q initially. The whole 8q chromosome arm became significant (using the center of masses) upon removal of two Luminal patients (110 and 302) with profiles that were clearly different from the rest of patients in the subtype. Similarly, 12q became significant (with TAaCGH) upon removal of the Luminal B Patient 378 (the patient already identified as an outlier in the Luminal B study). Our study using GISTIC found 4p, 5q, 10p, 10q, but failed to identify 6p, 15q.

ii. CNAs not reported in the original study, but validated in [32] using TAaCGH

**Figure 10:**
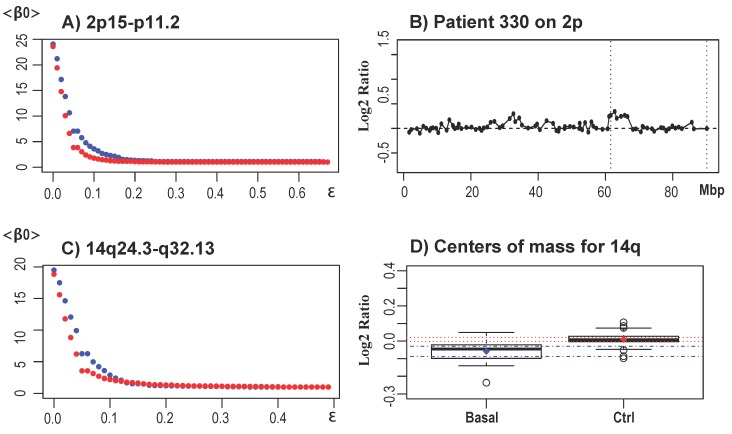
Results for the basal-like subtype. (**A**) $<\beta_{0}>$ curves for the significant region in the basal-like subtype 2p15 to p11.2. The blue curve corresponds to the basal-like subtype and the red curve to the control set. (**B**) A characteristic profile of a basal-like patient with an amplification at 2p14 to p13.3 (64 to 71 Mbp) in the significant region 2p15 to p11.2 (vertical dotted lines). (**C**) $<\beta_{0}>$ curves for significant region 14q24.3 to q32.13. The blue curve corresponds to the basal-like subtype and the red curve to the control set. (**D**) A box plot of the center of masses of the basal-like subtype *vs*. the control for 14q. Diamonds represent the average, and horizontal dotted lines represent the confidence intervals. Blue confidence intervals correspond to the basal-like subtype and red to the control set. The boxplot shows the displacement of the center of mass for basal-like in 14q as a full-length deletion.

We also detected regions that were validated in [32], but that were not reported in the original study [33]. These regions were 1p22.2 to p12, 1p36.32 to p31.1, 2p15 to p11.2 and most segments in 14q: 14q12 to q21.3 and 14q24.1 to q32.33. This region in 14q was large enough that the significance was also reflected in the analysis by the center of masses. Significant sections contained the cytobands 14q32 to q33 reported by [31,67], but partially missed the cytobands 14q22 to q23 reported by [32]. A detailed description of the significant clones in the basal-like subtype are presented in Table 3 and in Supplementary File S6. Figure 10 shows examples of the $<\beta_{0}>$ curves for chromosome arms 2p and 14q ((A) and (C)), a representative profile for chromosome 2p (B) and the displacement for the center of masses for 14q for the basal-like subtype (D). None of these regions were detected by our analysis using GISTIC.

iii. New CNAs not reported in the original study, not validated in [32], but confirmed by GISTIC

Some regions were not validated in [32], but were detected by GISTIC. We report these regions separately, because they have also been reported in other studies; hence, we do not believe they are an artifact of the data.

1. Chromosome arm 1q: We found the region 1q23.1 to 31.1 to be aberrant, and GISTIC confirmed it to be an amplification. This region was large enough that the changes were also detected in the study using the center of masses. This region of the genome was previously reported in other studies [31,32,36].
2. Chromosome arm 3p: Two regions in 3p were significant: 3p22.1 to p11.2 and 3p26.3 to p23. Region 3p22.1 to p11.2 has been reported to be a loss in a number of studies, including [32,68,69,70]. Additionally, we found a gain in 3p26.3 to p23.
3. Chromosome arm 3q: We found an amplification of the whole arm, while GISTIC found region 3q27.2. The gain of 3q is characteristic of BRCA1 deficiency in sporadic tumors, as well as in hereditary tumors (e.g., [71]).
4. Chromosome arm 6q: Three sections out of nine were found amplified in chromosome arm 6q. These three regions expanded cytobands 6q24.1 to q27 and contain the estrogen receptor gene ERS1 located at 6q25.2. This finding was also detected in our study by the center of masses [69].
5. Chromosome arm 12p: The region 12p13.3 was found to be amplified using our topological analysis, and the entire arm was also detected by the displacement of the center of masses. GISTIC detected a downstream region 12p33 to be amplified.
6. Chromosome arm 13q: Two main sections were found aberrant in the chromosome arm 13q. The first one expanding 13q12.2 to q31.2 and the second 13q31.2 to q34. We were unable to identify whether 13q12.2 to q31.2 was an amplification or a deletion; however, GISTIC identified a deletion in 13q14.11 in 81% of the patients. Additionally TAaCGH found an amplification in 13q31.2 to q34. Amplifications in chromosome 13q have been found in multiple subtypes [36] and more specifically in cytokeratin 14 (CK14) positive tumors, 25% of which are basal-like carcinomas [72].
7. Chromosome arm 18q: A section extending 18q11.1 to q21.33 was significant, but TAaCGH was unable to identify whether it was an amplification or a deletion, suggesting an heterogeneous combinations of amplifications and deletions in the region across subtypes. GISTIC, on the other hand, identified a deletion in 18q12.2.

**Table 3:** New chromosome aberrations detected by TAaCGH in the basal-like subtype.

Chromosome Arm	Cytoband	Location of Aberration	Gain/Loss	Location of Neighboring Clones
1p	36.21	14,639,539	loss	12,429,632 to 15,553,913
	35.1 to 32.3	34,380,537 to 53,737,606	gain	33,102,443 to 54,031,005
	32.3	55,875,826	loss	55,270,329 to 56,824,097
	32.2-31.3	58,882,124 to 62,359,674	gain	57,684,846 to 63,423,938
	31.1	78,005,143	gain	76,869,240 to 82,056,781
	22.1	93,996,738	loss	92,518,227 to 94,792,570
	21.2	99,899,192	gain	98,909,737 to 99,899,246
	21.1	101,303,797	loss	99,899,246 to 101,449,113
	21.2	101,449,113	gain	101,303,797 to 102,594,767
	13.3	107,585,795 to 108,529,492	gain	106,959,397 to 110,015,588
	13.1	117,424,118	gain	116,780,010 to 118,589,386
2p	14 to 13.3	64,625,779 to 71,023,979	gain	63,342,684 to 71,251,890
14q	13.2	33,766,983 to 36,544,890	loss	33,310,161 to 37,941,646
	21.1	37,941,646	loss	36,544,890 to 46,896,032
	21.2	44,219,870	loss	42,928,208 to 45,707,124
	24.3	76,584,595	loss	76,141,892 to 77,583,617
	31.1	80,271,909 to 83,099,727	loss	78,389,382 to 83,987,892
	31.2	84,784,824	loss	83,987,892 to 85,099,070
	31.3	87,007,447 to 87,344,554	loss	85,099,070 to 87,765,087
	31.3 to 32.12	89,750,111 to 92,321,034	loss	88,420,711 to 93,495,784
	32.13 to 32.2	94,877,177 to 97,996,975	loss	93,495,784 to 95,662,483


iv. New CNAs not reported in the original study, not validated in [32] using TAaCGH, but in agreement with other studies

Three chromosome arms were neither identified by GISTIC, nor validated in [32]. These arms, however, have been reported previously in other works.

1. Chromosome arm 4q: We found most of 4q to be significant, with the exception of the centromere near regions 4q11 to q13.3. This finding is in agreement with [32,69], who reported a loss of 4q31 to q35. The aberration in 4q was large enough to also be detected by the center of masses.
2. Chromosome arm 9p: Cytobands from 9p24.3 to 9p21.3 were found to be significant. Gains were reported in [63,73].
3. Chromosome arm Xp. The section containing Xp22.33 to 11.21, which contained all clones in the array, was found significant in [72].

v. New CNAs identified by TAaCGH, but not validated in [32]

Some regions were found to be significant only on the original dataset. These included section 5p15.33 to p12 and were also significant when considering the whole chromosome arm 5p and sections 9q21.13 to q22.32 and 9q32 to q34.3. Since these regions were not validated and have scarcely been investigated previously, we cataloged them as artifacts of the data.

vi. CNAS identified by GISTIC alone

GISTIC identified four chromosome arms that neither TAaCGH, nor SIRAC identified. There regions were an amplification in 7q34, a deletion in 8p23.2, a deletion in 11q24.3 and an amplification in 17q24.3. As discussed earlier, these regions were relatively common across different subtypes (see Supplementary File S7).

## 4. Conclusions

Array CGH provides an unparalleled opportunity to characterize disease-associated CNAs. Identification of these aberrations, however, is a difficult task, due to the heterogeneity of the diseases and the noise inherent to the microarray technologies. Here, we have presented a method called topological analysis of array CGH (TAaCGH), which complements currently-available methods. Other topological methods have been used in the identification of breast cancer subtypes using gene expression [74]; however, TAaCGH is, to our knowledge, the only supervised method that uses topological techniques to identify regions of copy number changes. In comparison to other methods that identify CNAs, TAaCGH incorporates a multi-resolution segmentation approach, modulated by the filtration parameter, and performs an association test between the topological properties of the point cloud and the phenotype under study.

TAaCGH is designed to analyze any type of time series provided that it is made of real-valued data. In previous works, we applied preliminary versions of TAaCGH to a different breast cancer aCGH dataset [22] and to a gene expression dataset [48], and in the future, we intend to extend it to other genomic data (see, for instance, [75]). Applications of TAaCGH to SNPs or sequencing data remain to be explored. In this study, we have extended our previous work by analyzing the statistical properties of TAaCGH, developing a new method to optimize the parameters in the program (specificity and sensitivity, dimensionality, size of the genomic section to be analyzed, comparison of test and control with CNAs at identical locations), identifying amplifications and deletions of specific clones within significant $<\beta_{0}>$ sections and incorporating the analysis of the center of masses for identifying whole arm amplifications/deletions.

We found almost all aberrations reported in the initial study [33] and 31 more regions that were missed in the original study. We validated many of the regions by testing TAaCGH on a second dataset [32] and by comparing our results with those obtained by GISTIC. Although the gene expression data in [33] was not made available to us, we were able to find some possible significance to the regions found using the database Catalogue of Somatic Mutations in Cancer (COSMIC) [76]. For instance, we found 11q22.1 to 23.2 in Luminal A. This aberration was not found in the original study by Horlings, was not validated in the dataset of [32], but was confirmed by GISTIC. Interestingly, 11q22.1 to q23.2 corresponds to the position of the progesterone receptor (PR) whose overexpression is commonly associated with Luminal A tumors. Supporting this finding, although not conclusive, we found a significant association between patients with an amplification in 11q22.1 to 23.2 and the reported PR status (Fischer exact test p=0.03). This result suggests that the PR gene can be regulated by changes in copy number.

We found significant aberrations on the Luminal B subtype only upon removal of the basal-like subtype from the control set. Besides those aberrations that were reported in the original study (*i.e.*, 1p36.32 to 31.1 and 8p23.3 to 23.1) [33] we also found the following: (1) Region 4q24 to q27 for which we did not find a specific amplification or deletion common to all patients, suggesting an heterogeneous pattern of amplification and deletions across the profiles. Inspection of the profiles revealed a large deletion in some patients [77] combined with a focal gain between positions 112,097,383 and 116,102,599. Analysis of the COSMIC database of these cytobands found gene UGT8 associated with malignancy and lung metastasis [78,79]. (2) Region 8p12 contains genes whose loss has been associated with breast cancer progression (*KAT6A, PURG, WRN, NRG1*) [80,81,82,83]. (3) 9p was driven by a combination of deletions and amplifications. We found a gain at 6,004,718 in 9p24.1, a region that contains multiple proto-oncogenes related to breast cancer (*i.e.*, GASC1 UHRF2, KIAA1432 and C9orf123) [63], as well as losses in positions 9,668,611, a region that contains the single gene PTPRD that has been associated with poor prognosis and metastasis in cancer [84,85]. Furthermore, the region 9p21.1 to q23 has been reported to be lost in breast cancer [86]. (4) We also found large deleted regions in chromosomes 9q and 13q, which contain multiple cancer genes, together with an amplification in 9q31.1. This amplification contains the gene SMC2, which has been associated with poor prognosis [87]. (5) A gain at 43,635,239 in 21q22.3 was also found. Our study did not find 17q. We believe that this was the result of having a small sample size for the Luminal B subtype and of having several patients in the control group with aberrations in the same region.

Basal-like tumors revealed a wide variety of aberrations, and in our study, we found all aberrations reported in the original study (chromosome arms 8q and 12q were found by removing three patients with very high copy number values; see Supplementary File S8) and 19 more aberrations. Out of these 19 aberrations, seven were also confirmed by GISTIC, and three were validated in a second independent dataset. Most of the others had been reported in other studies. These three new CNAs were found in chromosome arms 1p, 2p and 14q and are described in Table 3. Most significantly, we found intermittent regions of gains and losses between 1p36.32 and 1p13.1, a gain of 2p15 to p11.2 and losses of 14q12 to q21.3 and 14q24.3 to q32.22. We were unable to obtain any meaningful information from the COSMIC database, however, because these regions are large and dense in cancer genes.

In conclusion, topological approaches provide an alternative method for data analysis, and in this work, we have shown how it can help uncover chromosome aberrations in aCGH data. One important feature of this topological approach is that it can be extended in multiple directions. For instance, the work of Perea and Harer [23] and our own work [88,89] suggest that the number of two-dimensional holes in the data (denoted by $\beta_{1}$) can be used to identify periodic patterns in the data. We are currently working on such an analysis, but are unable to provide any results yet, since a new statistical framework needs to be developed. Our preliminary studies suggest that certain co-occurring aberrations can be detected using this topological invariant. Other possible extensions include different strategies in the generation of the point cloud (instead of a delay time embedding algorithm) or the incorporation of non-euclidean measures in the definition of the point cloud.

## 5. Software

TAaCGH can be obtained by e-mailing Javier Arsuaga directly: jarsuaga@ucdavis.edu.

## Supplementary Materials

Supplementary materials can be found at http://www.mdpi.com/2076-3905/4/3/339/s1.

## References

[1] Hanahan D., Weinberg R.A. (2011). Hallmarks of cancer: the next generation. Cell.

[2] Lopez-Garcia M.A., Geyer F.C., Lacroix-Triki M., Marchió C., Reis-Filho J.S. (2010). Breast cancer precursors revisited: molecular features and progression pathways. Histopathology.

[3] Kwei K.A., Kung Y., Salari K., Holcomb I.N., Pollack J.R. (2010). Genomic instability in breast cancer: Pathogenesis and clinical implications. Mol. Oncol..

[4] Bell D.W. (2010). Our changing view of the genomic landscape of cancer. J. Pathol..

[5] Mahmood S.F., Gruel N., Chapeaublanc E., Lescure A., Jones T., Reyal F., Vincent-Salomon A., Raynal V., Pierron G., Perez F. (2014). A siRNA screen identifies RAD21, EIF3H, CHRAC1 and TANC2 as driver genes within the 8q23, 8q24.3 and 17q23 amplicons in breast cancer with effects on cell growth, survival and transformation. Carcinogenesis.

[6] Wang E. (2013). Understanding genomic alterations in cancer genomes using an integrative network approach. Cancer Lett..

[7] Climent J., Dimitrow P., Fridlyand J., Palacios J., Siebert R., Albertson D.G., Gray J.W., Pinkel D., Lluch A., Martinez-Climent J.A. (2007). Deletion of chromosome 11q predicts response to anthracycline-based chemotherapy in early breast cancer. Cancer Res..

[8] Climent J., Garcia J., Mao J., Arsuaga J., Perez-Losada J. (2007). Characterization of breast cancer by array comparative genomic hybridization. This paper is one of a selection of papers published in this Special Issue, entitled 28th International West Coast Chromatin and Chromosome Conference, and has undergone the Journal’s usual peer review process. Biochem. Cell Biol..

[9] Desmedt C., Voet T., Sotiriou C., Campbell P.J. (2012). Next generation sequencing in breast cancer: First take home messages. Curr. Opin. Oncol..

[10] Doyle M.A., Li J., Doig K., Fellowes A., Wong S.Q. (2014). Studying Cancer Genomics Through Next-Generation DNA Sequencing and Bioinformatics. Clinical Bioinformatics.

[11] Meyerson M., Gabriel S., Getz G. (2010). Advances in understanding cancer genomes through second-generation sequencing. Nat. Rev. Genet..

[12] Pinkel D., Albertson D.G. (2005). Array comparative genomic hybridization and its applications in cancer. Nat. Genet..

[13] Beroukhim R., Mermel C.H., Porter D., Wei G., Raychaudhuri S., Donovan J., Barretina J., Boehm J.S., Dobson J., Urashima M. (2010). The landscape of somatic copy-number alteration across human cancers. Nature.

[14] Bignell G.R., Greenman C.D., Davies H., Butler A.P., Edkins S., Andrews J.M., Buck G., Chen L., Beare D., Latimer C. (2010). Signatures of mutation and selection in the cancer genome. Nature.

[15] Boyle J., Kreisberg R., Bressler R., Killcoyne S. (2012). Methods for visual mining of genomic and proteomic data atlases. BMC Bioinform..

[16] Ding L., Wendl M.C., McMichael J.F., Raphael B.J. (2014). Expanding the computational toolbox for mining cancer genomes. Nat. Rev. Genet..

[17] Fridlyand J., Snijders A.M., Pinkel D., Albertson D.G., Jain A.N. (2004). Hidden Markov models approach to the analysis of array CGH data. J. Multivar. Anal..

[18] Hupé P., Stransky N., Thiery J.P., Radvanyi F., Barillot E. (2004). Analysis of array CGH data: From signal ratio to gain and loss of DNA regions. Bioinformatics.

[19] Klijn C., Holstege H., de Ridder J., Liu X., Reinders M., Jonkers J., Wessels L. (2008). Identification of cancer genes using a statistical framework for multiexperiment analysis of nondiscretized array CGH data. Nucleic Acids Res..

[20] De Ronde J.J., Klijn C., Velds A., Holstege H., Reinders M.J., Jonkers J., Wessels L.F. (2010). KC-SMARTR: An R package for detection of statistically significant aberrations in multi-experiment aCGH data. BMC Res. Notes.

[21] Lai C., Horlings H.M., van de Vijver M.J., van Beers E.H., Nederlof P.M., Wessels L.F., Reinders M.J. (2007). SIRAC: Supervised Identification of Regions of Aberration in aCGH datasets. BMC Bioinform..

[22] De Woskin D., Climent J., Cruz-White I., Vazquez M., Park C., Arsuaga J. (2010). Applications of computational homology to the analysis of treatment response in breast cancer patients. Topol. Appl..

[23] Perea J.A., Harer J. (2013). Sliding windows and persistence: An application of topological methods to signal analysis. Found. Comput. Math..

[24] Edelsbrunner H., Harer J. (2008). Persistent homology-a survey. Contemp. Math..

[25] Zomorodian A.J. (2005). Topology for computing.

[26] Hsu L., Self S.G., Grove D., Randolph T., Wang K., Delrow J.J., Loo L., Porter P. (2005). Denoising array-based comparative genomic hybridization data using wavelets. Biostatistics.

[27] Sorlie T., Perou C.M., Tibshirani R., Aas T., Geisler S., Johnsen H., Hastie T., Eisen M., van de Rijn M., Jeffrey S. (2001). Gene expression patterns of breast carcinomas distinguish tumor subclasses with clinical implications. Proc. Natl. Acad. Sci. USA.

[28] Sørlie T., Tibshirani R., Parker J., Hastie T., Marron J., Nobel A., Deng S., Johnsen H., Pesich R., Geisler S. (2003). Repeated observation of breast tumor subtypes in independent gene expression data sets. Proc. Natl. Acad. Sci. USA.

[29] Perou C.M., Børresen-Dale A.L. (2011). Systems biology and genomics of breast cancer. Cold Spring Harb. Perspect. Biol..

[30] Shiu K.K., Natrajan R., Geyer F.C., Ashworth A., Reis-Filho J.S. (2010). DNA amplifications in breast cancer: Genotypic-phenotypic correlations. Future Oncol..

[31] Adélaïde J., Finetti P., Bekhouche I., Repellini L., Geneix J., Sircoulomb F., Charafe-Jauffret E., Cervera N., Desplans J., Parzy D. (2007). Integrated profiling of basal and luminal breast cancers. Cancer Res..

[32] Bergamaschi A., Kim Y.H., Wang P., Sørlie T., Hernandez-Boussard T., Lonning P.E., Tibshirani R., Børresen-Dale A.L., Pollack J.R. (2006). Distinct patterns of DNA copy number alteration are associated with different clinicopathological features and gene-expression subtypes of breast cancer. Genes Chromosomes Cancer.

[33] Horlings H.M., Lai C., Nuyten D.S., Halfwerk H., Kristel P., van Beers E., Joosse S.A., Klijn C., Nederlof P.M., Reinders M.J. (2010). Integration of DNA copy number alterations and prognostic gene expression signatures in breast cancer patients. Clin. Cancer Res..

[34] Jönsson G., Staaf J., Vallon-Christersson J., Ringnér M., Holm K., Hegardt C., Gunnarsson H., Fagerholm R., Strand C., Agnarsson B.A. (2010). Research article Genomic subtypes of breast cancer identified by array-comparative genomic hybridization display distinct molecular and clinical characteristics. Breast Cancer Res..

[35] Loo L.W., Grove D.I., Williams E.M., Neal C.L., Cousens L.A., Schubert E.L., Holcomb I.N., Massa H.F., Glogovac J., Li C.I. (2004). Array comparative genomic hybridization analysis of genomic alterations in breast cancer subtypes. Cancer Res..

[36] Cancer Genome Atlas Network (2012). Comprehensive molecular portraits of human breast tumours. Nature.

[37] Bilal E., Vassallo K., Toppmeyer D., Barnard N., Rye I.H., Almendro V., Russnes H., Børresen-Dale A.L., Levine A.J., Bhanot G. (2012). Amplified loci on chromosomes 8 and 17 predict early relapse in ER-positive breast cancers. PLoS ONE.

[38] Climent J., Martinez-Climent J.A., Blesa D., Garcia-Barchino M.J., Saez R., Sánchez-Izquierdo D., Azagra P., Lluch A., Garcia-Conde J. (2002). Genomic loss of 18p predicts an adverse clinical outcome in patients with high-risk breast cancer. Clin. Cancer Res..

[39] Han W., Han M.R., Kang J.J., Bae J.Y., Lee J.H., Bae Y.J., Lee J.E., Shin H.J., Hwang K.T., Hwang S.E. (2006). Genomic alterations identified by array comparative genomic hybridization as prognostic markers in tamoxifen-treated estrogen receptor-positive breast cancer. BMC Cancer.

[40] Hwang K.T., Han W., Cho J., Lee J.W., Ko E., Kim E.K., Jung S.Y., Jeong E.M., Bae J.Y., Kang J.J. (2008). Genomic copy number alterations as predictive markers of systemic recurrence in breast cancer. Int. J. Cancer.

[41] McDonald S.L., Stevenson D.A., Moir S.E., Hutcheon A.W., Haites N.E., Heys S.D., Schofield A.C. (2005). Genomic changes identified by comparative genomic hybridisation in docetaxel-resistant breast cancer cell lines. Eur. J. Cancer.

[42] Rouzier R., Perou C.M., Symmans W.F., Ibrahim N., Cristofanilli M., Anderson K., Hess K.R., Stec J., Ayers M., Wagner P. (2005). Breast cancer molecular subtypes respond differently to preoperative chemotherapy. Clin. Cancer Res..

[43] Seute A., Sinn H.P., Schlenk R.F., Emig R., Wallwiener D., Grischke E.M., Hohaus S., Döhner H., Haas R., Bentz M. (2001). Clinical relevance of genomic aberrations in homogeneously treated high-risk stage II/III breast cancer patients. Int. J. Cancer.

[44] Sun Y., Urquidi V., Goodison S. (2010). Derivation of molecular signatures for breast cancer recurrence prediction using a two-way validation approach. Breast Cancer Res. Treat..

[45] Turner N., Lambros M.B., Horlings H.M., Pearson A., Sharpe R., Natrajan R., Geyer F.C., van Kouwenhove M., Kreike B., Mackay A. (2010). Integrative molecular profiling of triple negative breast cancers identifies amplicon drivers and potential therapeutic targets. Oncogene.

[46] Lai W.R., Johnson M.D., Kucherlapati R., Park P.J. (2005). Comparative analysis of algorithms for identifying amplifications and deletions in array CGH data. Bioinformatics.

[47] Troyanskaya O., Cantor M., Sherlock G., Brown P., Hastie T., Tibshirani R., Botstein D., Altman R.B. (2001). Missing value estimation methods for DNA microarrays. Bioinformatics.

[48] Arsuaga J., Baas N.A., DeWoskin D., Mizuno H., Pankov A., Park C. (2012). Topological analysis of gene expression arrays identifies high risk molecular subtypes in breast cancer. Appl. Algebra Eng. Commun. Comput..

[49] Sexton H., Vejdemo-Johansson M. jPlex, 2008. http://comptop.stanford.edu/programs/jplex/.

[50] Benjamini Y., Hochberg Y. (1995). Controlling the false discovery rate: A practical and powerful approach to multiple testing. J. R. Stat. Soc. Series B Stat. Methodol..

[51] Storey J.D., Tibshirani R. (2003). Statistical significance for genomewide studies. Proc. Natl. Acad. Sci..

[52] Chin K., DeVries S., Fridlyand J., Spellman P.T., Roydasgupta R., Kuo W.L., Lapuk A., Neve R.M., Qian Z., Ryder T. (2006). Genomic and transcriptional aberrations linked to breast cancer pathophysiologies. Cancer Cell.

[53] Olshen A.B., Venkatraman E., Lucito R., Wigler M. (2004). Circular binary segmentation for the analysis of array-based DNA copy number data. Biostatistics.

[54] Ades F., Zardavas D., Bozovic-Spasojevic I., Pugliano L., Fumagalli D., de Azambuja E., Viale G., Sotiriou C., Piccart M. (2014). Luminal B breast cancer: Molecular characterization, clinical management, and future perspectives. J. Clin. Oncol..

[55] Fridlyand J., Snijders A.M., Ylstra B., Li H., Olshen A., Segraves R., Dairkee S., Tokuyasu T., Ljung B.M., Jain A.N. (2006). Breast tumor copy number aberration phenotypes and genomic instability. BMC Cancer.

[56] Natrajan R., Lambros M.B., Geyer F.C., Marchio C., Tan D.S., Vatcheva R., Shiu K.K., Hungermann D., Rodriguez-Pinilla S.M., Palacios J. (2009). Loss of 16q in high grade breast cancer is associated with estrogen receptor status: Evidence for progression in tumors with a luminal phenotype?. Genes Chromosomes Cancer.

[57] Bièche I., Khodja A., Lidereau R. (1999). Deletion mapping of chromosomal region 1p32-pter in primary breast cancer. Genes Chromosomes Cancer.

[58] Chin S., Wang Y., Thorne N., Teschendorff A., Pinder S., Vias M., Naderi A., Roberts I., Barbosa-Morais N., Garcia M. (2007). Using array-comparative genomic hybridization to define molecular portraits of primary breast cancers. Oncogene.

[59] Ray M.E., Yang Z.Q., Albertson D., Kleer C.G., Washburn J.G., Macoska J.A., Ethier S.P. (2004). Genomic and expression analysis of the 8p11–12 amplicon in human breast cancer cell lines. Cancer Res..

[60] Streicher K., Yang Z., Draghici S., Ethier S. (2007). Transforming function of the LSM1 oncogene in human breast cancers with the 8p11–12 amplicon. Oncogene.

[61] Yang Z.Q., Albertson D., Ethier S.P. (2004). Genomic organization of the 8p11–p12 amplicon in three breast cancer cell lines. Cancer Genet. Cytogenet..

[62] Yang Z.Q., Streicher K.L., Ray M.E., Abrams J., Ethier S.P. (2006). Multiple interacting oncogenes on the 8p11–p12 amplicon in human breast cancer. Cancer Res..

[63] Wu J., Liu S., Liu G., Dombkowski A., Abrams J., Martin-Trevino R., Wicha M., Ethier S., Yang Z. (2012). Identification and functional analysis of 9p24 amplified genes in human breast cancer. Oncogene.

[64] Carlson R.W., Moench S.J., Hammond M., Perez E.A., Burstein H.J., Allred D.C., Vogel C.L., Goldstein L.J., Somlo G., Gradishar W.J. (2006). HER2 testing in breast cancer: NCCN Task Force report and recommendations. J. Natl. Compr. Cancer Netw..

[65] Wolff A.C., Hammond M.E.H., Hicks D.G., Dowsett M., McShane L.M., Allison K.H., Allred D.C., Bartlett J.M., Bilous M., Fitzgibbons P. (2013). Recommendations for human epidermal growth factor receptor 2 testing in breast cancer: American Society of Clinical Oncology/College of American Pathologists clinical practice guideline update. J. Clin. Oncol..

[66] Staaf J., Jonsson G., Ringner M., Vallon-Christersson J., Grabau D., Arason A., Gunnarsson H., Agnarsson B.A., Malmstrom P.O., Johannsson O.T., Loman N., Barkardottir R.B., Borg A. (2010). High-resolution genomic and expression analyses of copy number alterations in HER2-amplified breast cancer. Breast Cancer Res..

[67] Gao Y., Niu Y., Wang X., Wei L., Lu S. (2009). Genetic changes at specific stages of breast cancer progression detected by comparative genomic hybridization. J. Mol. Med..

[68] de Oliveira M.M.C., de Oliveira S.F.V., Lima R.S., de Andrade Urban C., Cavalli L.R., Ribeiro E.M.d.S.F., Cavalli I.J. (2012). Differential loss of heterozygosity profile on chromosome 3p in ductal and lobular breast carcinomas. Hum. Pathol..

[69] Fang M., Toher J., Morgan M., Davison J., Tannenbaum S., Claffey K. (2011). Genomic differences between estrogen receptor (ER)-positive and ER-negative human breast carcinoma identified by single nucleotide polymorphism array comparative genome hybridization analysis. Cancer.

[70] Qian P., Banerjee A., Wu Z.S., Zhang X., Wang H., Pandey V., Zhang W.J., Lv X.F., Tan S., Lobie P.E. (2012). Loss of SNAIL regulated miR-128-2 on chromosome 3p22.3 targets multiple stem cell factors to promote transformation of mammary epithelial cells. Cancer Res..

[71] Joosse S.A., Brandwijk K.I., Mulder L., Wesseling J., Hannemann J., Nederlof P.M. (2011). Genomic signature of BRCA1 deficiency in sporadic basal-like breast tumors. Genes Chromosomes Cancer.

[72] Jones C., Ford E., Gillett C., Ryder K., Merrett S., Reis-Filho J.S., Fulford L.G., Hanby A., Lakhani S.R. (2004). Molecular cytogenetic identification of subgroups of grade III invasive ductal breast carcinomas with different clinical outcomes. Clin. Cancer Res..

[73] Vincent-Salomon A., Gruel N., Lucchesi C., MacGrogan G., Dendale R., Sigal-Zafrani B., Longy M., Raynal V., Pierron G., de Mascarel I. (2007). Identification of typical medullary breast carcinoma as a genomic sub-group of basal-like carcinomas, a heterogeneous new molecular entity. Breast Cancer Res..

[74] Nicolau M., Levine A.J., Carlsson G. (2011). Topology based data analysis identifies a subgroup of breast cancers with a unique mutational profile and excellent survival. Proc. Natl. Acad. Sci..

[75] Steiner M.A. (2015). Classification of breast cancer subtypes using signaling pathways and persistent homology. Master’s thesis.

[76] Forbes S., Bhamra G., Bamford S., Dawson E., Kok C., Clements J., Menzies A., Teague J., Futreal P., Stratton M. (2008). The catalogue of somatic mutations in cancer (COSMIC). Curr. Protoc. Hum. Genet..

[77] Jönsson G., Naylor T.L., Vallon-Christersson J., Staaf J., Huang J., Ward M.R., Greshock J.D., Luts L., Olsson H., Rahman N. (2005). Distinct genomic profiles in hereditary breast tumors identified by array-based comparative genomic hybridization. Cancer Res..

[78] Dziegeil P., Owczarek T., Plazuk E., Gomułkiewicz A., Majchrzak M., Podhorska-Okołów M., Driouch K., Lidereau R., Ugorski M. (2010). Ceramide galactosyltransferase (UGT8) is a molecular marker of breast cancer malignancy and lung metastases. Br. J. Cancer.

[79] Ruckhäberle E., Karn T., Rody A., Hanker L., Gätje R., Metzler D., Holtrich U., Kaufmann M. (2009). Gene expression of ceramide kinase, galactosyl ceramide synthase and ganglioside GD3 synthase is associated with prognosis in breast cancer. J. Cancer Res. Clin. Oncol..

[80] Opresko P.L., Calvo J.P., von Kobbe C. (2007). Role for the Werner syndrome protein in the promotion of tumor cell growth. Mech. Ageing Dev..

[81] Pole J., Courtay-Cahen C., Garcia M., Blood K., Cooke S., Alsop A., Tse D., Caldas C., Edwards P. (2006). High-resolution analysis of chromosome rearrangements on 8p in breast, colon and pancreatic cancer reveals a complex pattern of loss, gain and translocation. Oncogene.

[82] Suhasini A.N., Brosh R.M. (2013). Disease-causing missense mutations in human DNA helicase disorders. Mutation Res..

[83] Turner-Ivey B., Guest S.T., Irish J.C., Kappler C.S., Garrett-Mayer E., Wilson R.C., Ethier S.P. (2014). KAT6A, a Chromatin Modifier from the 8p11–p12 Amplicon is a Candidate Oncogene in Luminal Breast Cancer. Neoplasia.

[84] Ortiz B., Fabius A.W., Wu W.H., Pedraza A., Brennan C.W., Schultz N., Pitter K.L., Bromberg J.F., Huse J.T., Holland E.C. (2014). Loss of the tyrosine phosphatase PTPRD leads to aberrant STAT3 activation and promotes gliomagenesis. Proc. Natl. Acad. Sci..

[85] Ortiz B., White J.R., Wu W.H., Chan T.A. (2014). Deletion of Ptprd and Cdkn2a cooperate to accelerate tumorigenesis. Oncotarget.

[86] An H.X., Claas A., Savelyeva L., Seitz S., Schlag P., Scherneck S., Schwab M. (1999). Two regions of deletion in 9p23–24 in sporadic breast cancer. Cancer Res..

[87] Wernicke M., Roitman P., Manfre D., Stern R. (2011). Breast cancer and the stromal factor. The prometastatic healing proces hypothesis. Medicina (B Aires).

[88] Cavalcante R. (2012). Using homology and networks to locate copy number aberrations associated to recurrence in breast cancer. Master’s thesis.

[89] Rebouh M. (2012). Exploring topological methods to study genomic imbalance in breast cancer. Master’s thesis.

