# Supplementary material for: Identification of Copy Number Aberrations in Breast Cancer Subtypes Using Persistence Topology
Source: Microarrays (Basel). 2015 Aug 12;4(3):339–69. doi: 10.3390/microarrays4030339 (PMC4996377; doi:10.3390/microarrays4030339)
Supplement: Supplementary file 1 [file microarrays-04-00339-s001.zip › microarrays-83555-SI/S8-Outliers.pdf]

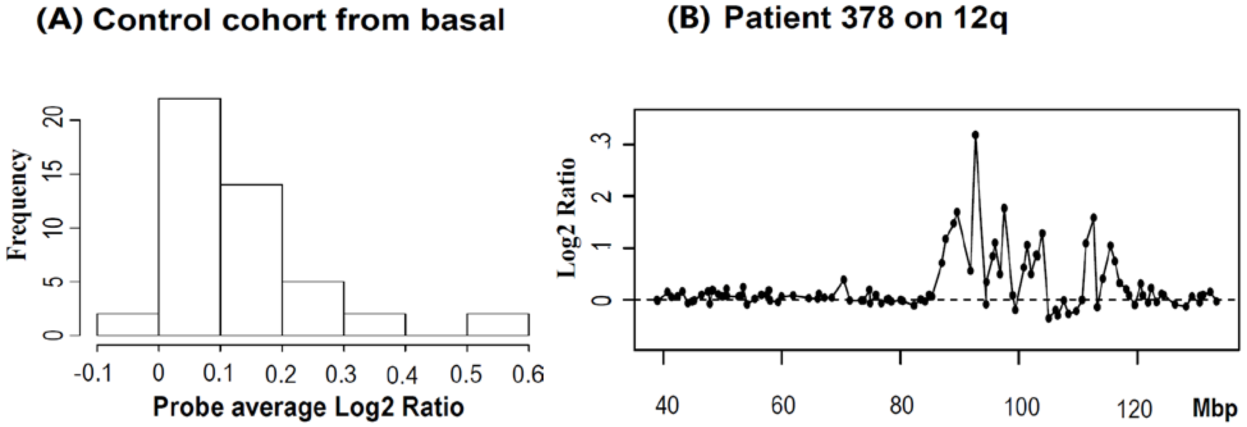

Supplementary File 8. (**A**) shows a histogram from the control cohort for Basal-like test on 8q. The bar at an average log-ratio of [0.5, 0.6] are the patients 110 and 302 which have a heavy weight when computing centers of mass; (**B**) shows an aCGH profile for patient 378 from luminal B cohort on 12q. The Y axis in this profile have a maximum of 3.5 when for every other patient in the data set was 1.7
